# Detecting Aquaporin Function and Regulation

**DOI:** 10.3389/fchem.2016.00003

**Published:** 2016-02-01

**Authors:** Ana Madeira, Teresa F. Moura, Graça Soveral

**Affiliations:** ^1^Research Institute for Medicines (iMed.ULisboa), Faculty of Pharmacy, Universidade de LisboaLisboa, Portugal; ^2^Faculdade de Ciências e Tecnologia, Universidade Nova de LisboaCaparica, Portugal; ^3^Departamento Bioquimica e Biologia Humana, Faculty of Pharmacy, Universidade de LisboaLisboa, Portugal

**Keywords:** aquaporin, aquaglyceroporin, membrane, water permeability, glycerol, channel, regulation, inhibition

## Abstract

Water is the major component of cells and tissues throughout all forms of life. Fluxes of water and solutes through cell membranes and epithelia are essential for osmoregulation and energy homeostasis. Aquaporins are membrane channels expressed in almost every organism and involved in the bidirectional transfer of water and small solutes across cell membranes. Aquaporins have important biological roles and have been implicated in several pathophysiological conditions suggesting a great translational potential in aquaporin-based diagnostics and therapeutics. Detecting aquaporin function is critical for assessing regulation and screening for new activity modulators that can prompt the development of efficient medicines. Appropriate methods for functional analysis comprising suitable cell models and techniques to accurately evaluate water and solute membrane permeability are essential to validate aquaporin function and assess short-term regulation. The present review describes established assays commonly used to assess aquaporin function in cells and tissues, as well as the experimental biophysical strategies required to reveal functional regulation and identify modulators, the first step for aquaporin drug discovery.

## Introduction

Water homeostasis and energy balance are essential for survival and adaptation of living cells. Water crosses cell membranes by two parallel pathways, with distinct mechanisms for permeation: partition/diffusion of water molecules across the hydrophobic bilayer and water diffusion through specialized protein channels known as aquaporins (Verkman, 2000). In either case, lipid or channel pathway, the driving force for water movement is the chemical potential of water (osmotic and/or hydrostatic pressure gradients) between both sides of the membrane. Compared to lipid bilayer diffusion, lower activation energy for transport is a typical feature of aquaporin-mediated diffusion.

Aquaporins (AQPs) belong to a highly conserved group of membrane proteins called the major intrinsic proteins that form a large family comprising more than 1700 integral membrane proteins found in virtually all-living organisms (Abascal et al., 2014). AQPs can be divided into three subfamilies: (i) orthodox or classical aquaporins, considered to be water selective, (ii) aquaglyceroporins, permeable to glycerol and other small solutes in addition to water, and (iii) S-aquaporins, also called unorthodox superaquaporins or subcellular aquaporins, a third subfamily only present in animals but not in plants, fungi and bacteria (Ishibashi et al., 2014) with permeability still uncertain.

The number of AQP isoforms expressed varies significantly among organisms. For instance, *Escherichia coli* possesses one classical AQP (AqpZ) and one AQP-like sequence (glycerol facilitator GlpF; Maurel et al., 1994; Calamita et al., 1995); the yeast *Saccharomyces cerevisiae* has two orthodox aquaporins (ScAqy1 and ScAqy2) and two aquaglyceroporins (YFL054Cp and ScFps1; Soveral et al., 2010) and plants possess up to 35 different isoforms (Maurel et al., 2008). In mammals 13 isoforms were identified (AQP0–12) and found differentially expressed in organs and tissues involved in fluid absorption or excretion but also in non-fluid transporting tissues like brain, skin, fat, and liver (Ishibashi et al., 2009).

The most remarkable feature of AQP channels is their high selectivity and efficiency on water or glycerol permeation, excluding ions, and protons (Murata et al., 2000). Apart from water and glycerol, a number of other permeants such as urea, ammonia, hydrogen peroxide, carbon dioxide, metalloids, nitric oxide (Wu and Beitz, 2007), and even ions (Yool and Campbell, 2012) were reported to permeate specific AQPs, although the mechanism of permeation is still obscure.

Regulation of AQPs is critical to osmoregulation and water homeostasis in microorganism and in mammalian organs involved in fluid transport (Kortenoeven and Fenton, 2014). Eukaryotic orthodox (water selective) AQPs are frequently regulated post-translationally either by gating, controlling the channels flux rate, or by trafficking, whereby AQPs are shuttled from intracellular storage sites to the plasma membrane (Törnroth-Horsefield et al., 2010). Gating of AQPs has been described for several cell systems. Factors like phosphorylation, pH, pressure, solute gradients, temperature, membrane tension among others, were reported to affect the gating behavior of yeast, plant and mammalian AQPs (Soveral et al., 1997a, 2008; Chaumont et al., 2005; Maurel, 2007; Törnroth-Horsefield et al., 2010; Leitao et al., 2012, 2014; Ozu et al., 2013).

Due to their unique ability to transport glycerol, AQPs play critical roles in osmoregulation by controlling the intracellular accumulation of glycerol. For example, yeast osmostress-induced glycerol accumulation is controlled by the high osmolarity glycerol (HOG) pathway at the level of gene expression, metabolism and transport. Regulation of the yeast aquaglyceroporin Fps1 that changes from open to closed state to ensure intracellular retention and accumulation of glycerol produced by alcohol fermentation is crucial for cells osmoprotective strategy (Ahmadpour et al., 2014).

In mammals, AQPs have also important roles in energy metabolism. By controlling glycerol content in epidermal, fat and other tissues, aquaglyceroporins are involved in skin hydration, cell proliferation, carcinogenesis and fat metabolism (Hara-Chikuma and Verkman, 2006; Rodríguez et al., 2011; Ribatti et al., 2014). Glycerol permeability in membranes from various tissues and organs has a key role in the regulation of metabolic and energy homeostasis, with the adipose tissue having a pivotal role (Madeira et al., 2015; Rodriguez et al., 2015). Whereas adipose aquaglyceroporin expression is hormone-mediated, triggered by catecholamines and insulin in fasting or feeding situations (Fruhbeck et al., 2014), less is known about their short-term regulation or gating.

Additionally in recent years multiple compounds have been described as inhibitors of AQPs water transport activity, but only a limited number was described for glycerol permeation via aquaglyceroporins (de Almeida et al., 2014). AQPs based modulator drugs are predicted to be of broad utility in the treatment of several disorders, such as cerebral edema, cancer, obesity, wound healing, epilepsy, glaucoma, and malaria (de Almeida et al., 2014; Verkman et al., 2014).

This review summarizes the biophysical approaches most frequently used to detect aquaporin activity in tissue and cell membranes and describes the experimental strategies required to uncover functional regulation and screen for chemical modulators.

## Cell models for functional analysis

Due to the widespread distribution of AQPs in nature, water transport assays have been performed using isolated cells from different organisms, such as bacteria (Delamarche et al., 1999; Mallo and Ashby, 2006), yeast (Soveral et al., 2007; Madeira et al., 2010), and mammalian cells (Solenov et al., 2004; Madeira et al., 2013, 2014a). Intracellular vesicles (Coury et al., 1999; Meyrial et al., 2001) as well as plasma membrane vesicles obtained from animal tissues (mainly kidney or intestinal epithelia) have been used to evaluate AQP activity either in intracellular organelles (Calamita et al., 2005; Noronha et al., 2014) or through epithelial membranes (apical or basolateral; Verkman et al., 1985; Soveral et al., 1997b; Mollajew et al., 2010). Another widely used approach to functionally characterize novel AQP isoforms consists in AQP heterologous expression in *Xenopus laevis* oocytes, which have very low intrinsic water permeability (Preston et al., 1992). In addition to oocytes, yeast cells lacking endogenous AQPs have been used to detect water transport capacity of mammalian (Pettersson et al., 2006) and plant (Leitao et al., 2012) aquaporins and to develop a generic high-throughput assay to identify functional AQP mutants resistant to freeze-thawing challenge (To et al., 2015). AQP-transfected cell lines (Ma et al., 1993) and mRNA injected Zebrafish embryos (Ikeda et al., 2011) have also been used for heterologous expression.

AQPs from different organisms have also been purified and reconstituted in liposomes, which allowed establishing their direct role in water/solute transport (van Hoek and Verkman, 1992; Zeidel et al., 1992). Phenotypic analysis of transgenic mice lacking AQPs has also brought new insights into their mechanisms of permeation and revealed their involvement in multiple biological functions, including transepithelial fluid transport, cell migration, brain edema, neuroexcitation, cell proliferation, epidermal water retention, and adipocyte metabolism (Verkman et al., 2014).

## Permeability assays

**Figure 1:**
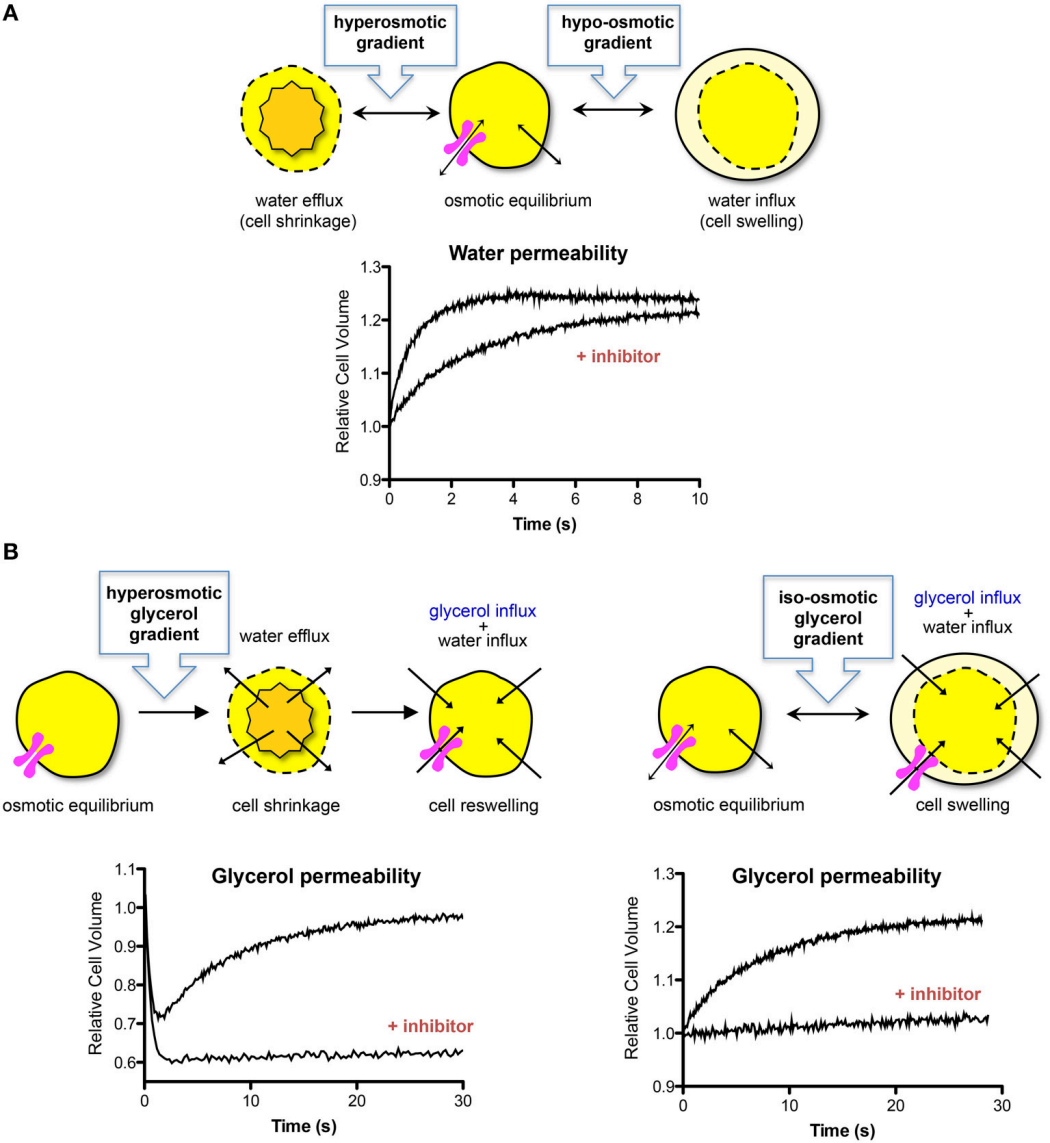
**Water and solute transport across biological membranes. (A)** Cell volume changes due to water fluxes after an imposed hyperosmotic or hypo-osmotic gradient with an impermeant solute. Water crosses cell membranes simultaneously through the lipid bilayer and AQPs inducing cell shrinkage or swelling, until a new osmotic equilibrium is reached. The presence of functional AQPs increases the rate of cell volume change. The graph shows a typical stopped-flow signal where the rate of volume change after a hypo-osmotic shock induces cell swelling. In the presence of an AQP inhibitor, the rate of swelling is decreased. **(B)** Cell volume change after imposing a glycerol gradient to cells expressing functional aquaglyceroporins. Left: hyperosmotic glycerol gradient. After the first fast cell shrinkage due to water outflow, glycerol influx in response to its chemical gradient is followed by water influx with subsequent cell reswelling. The graph shows a typical stopped-flow signal where the rate of volume change after imposing a glycerol gradient is biphasic, corresponding to the first fast water efflux followed by glycerol uptake and cell volume recover. In the presence of an aquaglyceroporin inhibitor, the rate of reswelling is strongly reduced. Right: iso-osmotic glycerol gradient. Glycerol influx due to its chemical gradient is followed by water influx with consequent cell swelling. The graph shows a typical stopped-flow signal where the rate of volume change after imposing a glycerol gradient induces cell swelling. An aquaglyceroporin inhibitor drastically reduces the rate of swelling.

Measurements of water or solute permeability of biological membranes indirectly indicate AQP expression and functional status. Regardless of the biological model used, the characterization of AQP activity and evaluation of water permeability is centered on following cell and/or vesicle volume changes resulting directly from water fluxes driven by osmotic and/or pressure gradients (Figure 1A). On the other hand, for the estimation of solute permeability, both the solute fluxes (driven by solute gradients) and the water fluxes have to be taken into account when analyzing the volume changes (Verkman et al., 2000; Figure 1B). Both the water and the solute fluxes are directly proportional to their respective driving forces, with the proportionality constants being the osmotic permeability (*P*_*f*_) and the solute permeability (*P*_*s*_) coefficient, respectively. The rate at which the volume changes occur depends on the fraction of water/or solute that permeates the channel (aqueous pathway) vs. the lipid bilayer diffusion. In addition, evaluating the permeability along temperature allows computing the activation energy (*E*_*a*_) for transport, a valuable parameter to detect aquaporin functional activity. Water or solute fluxes through a hydrophilic channel pore need lower activation energy *E*_*a*_ than fluxes across a hydrophobic lipid bilayer, and thus high permeability and low *E*_*a*_ indicate permeation via AQPs. An overview on the equations and parameters used to evaluate membrane water permeability can be found in Soveral et al. (2010).

Techniques to evaluate permeability make use of volume-dependent optical properties, such as light transmission (Farinas and Verkman, 1996; Farinas et al., 1997), absorbance (Levin et al., 2007), or scattering (Verkman et al., 1985; Soveral et al., 1997b, 2008) and fluorescence (Hamann et al., 2002; Solenov et al., 2004; Soveral et al., 2007). Equivalent strategies are also commonly used to assess the kinetics of transport in cells and proteoliposomes. Combining different biological models (cells/vesicles/proteoliposomes) and optical detection systems offers many possibilities to conduct research on AQP function. The most commonly used approaches will be further discussed.

### Epithelial assays

Water permeability may be determined in native epithelial tissues (e.g., intestinal wall, kidney tubules) or in epithelial cell monolayers cultured on permeable supports and mounted on Ussing chambers (Clarke, 2009). In this experimental setting, apical and basolateral membranes of polarized cells face different compartments. By adding a membrane impermeant solute such as sucrose or mannitol to one compartment, the generated transepithelial water flux is measured by the height of the fluid in a capillary tube connected to the other compartment (Dorr et al., 1997). Alternatively, a fluorescent dye is added to the hyperosmotic compartment and the rate of fluorescence change due to dye dilution is used to evaluate the total transepithelial osmotic water permeability (Levin et al., 2006). The total transepithelial permeability is the sum of two pathways, cellular and paracellular, where the presence of AQPs only affects the cellular pathway. The individual membrane permeability from these bipolar epithelial cells can be evaluated using isolated vesicles from either basolateral or apical membranes. This approach is widely used for AQP identification and characterization in epithelial membranes (Verkman et al., 1985; Soveral et al., 1997b; Alves et al., 1999).

### Osmotic swelling assays

AQP-mediated water transport can be evaluated by heterologous expression in *X. laevis* oocytes using an osmotic swelling assay (Preston et al., 1992; Verkman, 2000). Oocytes microinjected with AQP mRNA are subjected to hypo-osmotic gradients and the time course of cell swelling is followed by video microscopy. To test for solute permeability, an inwardly directed solute gradient is imposed, resulting in solute influx in response to the generated chemical gradient, followed by water influx and consequent oocyte swelling (Beitz et al., 2009). This system is particularly advantageous for studying AQPs due to oocytes endogenous low water permeability and hardly detectable glycerol and other solutes permeability.

A similar swelling assay uses erythrocytes expressing endogenous AQPs. Human erythrocytes express only one aquaglyceroporin isoform, AQP3. After challenged with hyperosmotic solute (glycerol) gradients, glycerol influx causes erythrocyte swelling and eventually cell hemolysis that can be monitored as a decrease in light absorption at 625 nm (Campos et al., 2011). The rate constant of hemolysis can be used to calculate glycerol permeability.

### Microscopy techniques

Over the years many microscopy techniques have been employed to characterize AQP function, namely phase contrast (Preston et al., 1992), dark field/phase contrast (Farinas et al., 1997), interferometry (Farinas and Verkman, 1996), confocal (Zelenina and Brismar, 2000), and fluorescence microscopy (Verkman, 2000; Solenov et al., 2004; Madeira et al., 2013).

The most currently used microscopy approaches exploit the properties of volume sensitive fluorescent dyes that undergo intracellular de-esterification and trapping. Cells are loaded with the membrane-permeant non-fluorescent precursor (e.g., calcein acetoxymethyl ester), which is cleaved intracellularly by non-specific esterases yielding the impermeable fluorescent form that gets trapped inside the cells (Madeira et al., 2013). Changes in fluorescence intensity resulting from osmotically induced volume changes can be monitored as the fluorescence of the fluophore is quenched. Two hypotheses explain this phenomenon inside the cell: (1) the quenching is mediated by cytoplasmic proteins, whose concentrations change as cells shrink or swell (Solenov et al., 2004); (2) fluorophores undergo self-quenching, i.e., fluorescence intensity decreases with increasing fluorophore concentration (Hamann et al., 2002). In any case, water and/or solutes permeability can be inferred from the linear relationship between cell volume and dye fluorescence intensity (Hamann et al., 2002).

Another method uses a genetically encoded optical sensor (yellow fluorescent protein YFP-H148Q-V163S), whose fluorescence is quenched by chloride (Galietta et al., 2001; Baumgart et al., 2012). Cell volume changes and consequent altered intracellular chloride concentration results in altered YFP emitted fluorescence.

More recently, a high throughput system for automated water/solute permeability measurements using volume-sensitive fluorescent indicators has been optimized taking advantage of microplate readers (Fenton et al., 2010).

### Stopped-flow spectroscopy

The stopped flow technique is the leading method to follow the fast-kinetics of cell volume change after a rapidly imposed osmotic/solute gradient. It has been frequently used to measure permeability of cell suspensions (Ma et al., 1993; Dobbs et al., 1998; Soveral et al., 2006, 2007; Martins et al., 2012), vesicles (Verkman et al., 1985; Soveral et al., 1997b; Alves et al., 1999), and proteoliposomes (van Hoek and Verkman, 1992; Zeidel et al., 1992; Noronha et al., 2014).

In the stopped flow device, cell/vesicle suspensions are subjected to osmotic challenges by rapid mixing with an equal volume of hypo- or hyper-osmotic solution (Figure 1). Osmotic water fluxes produce changes in cell volume, with consequent alterations in scattered light intensity or in fluorescence if the cells/vesicles are loaded with volume sensitive fluorescent dyes (e.g., fluorescein or calcein). Due to a linear relation between the optical properties of the system and cell volume, water, or solute movements can be followed until osmotic equilibrium is attained. Analysis of the relative volume changes due to osmotic or solute gradients allows quantification of osmotic permeability (*P*_*f*_) or solute permeability (*P*_*s*_) coefficients. Figure 1 displays the possible experimental configurations for measuring membrane water and glycerol permeability using the stop-flow technique.

### Computational methods

The introduction of computational methods has shed new light on AQPs water and solute permeation mechanisms. These methods make use of the available high-resolution atomic structures for different AQP isoforms (Fu et al., 2000; Sui et al., 2001; Savage et al., 2003; Harries et al., 2004; Lee et al., 2005; Törnroth-Horsefield et al., 2006; Horsefield et al., 2008; Newby et al., 2008; Fischer et al., 2009; Ho et al., 2009; Frick et al., 2014). Specifically, molecular dynamics simulations, which employ classical mechanics for the sampling of conformational changes in biomolecules, provided a unique dynamic insight onto AQPs structures (Hub et al., 2009). In the absence of a solved atomic structure for a specific AQP isoform, it is possible to assemble three-dimensional models using experimentally determined structures of related family members as templates (Bordoli et al., 2009). The assembly of homology models, having GlpF channel as a template, allowed accessing the structural uniqueness of human AQP3 (Martins et al., 2012), AQP7 (Madeira et al., 2014b), and AQP9 (Wacker et al., 2013).

## Assessing AQP inhibition and short-term regulation

Short-term regulation of AQPs, also known as gating, is often achieved by mechanisms directly affecting the protein channel conformation, which in turn impacts its transport activity (Alleva et al., 2012). It has been reported gating of eukaryotic AQPs by pH (Nemeth-Cahalan and Hall, 2000; Leitao et al., 2012), phosphorylation (Maurel et al., 1995; Fushimi et al., 1997; Kitchen et al., 2015), divalent cations (Zelenina et al., 2003), and membrane stretching (membrane surface tension; Soveral et al., 1997a, 2008; Ozu et al., 2013; Leitao et al., 2014). A comprehensive review on the therapeutic modulation of aquaporin functionality can be found in Beitz et al. (2015).

*X. laevis* oocytes swelling assays were the first used to show inhibition of AQP1 by mercury chloride (Preston et al., 1992) and are frequently used to detect AQP inhibition (Detmers et al., 2006; Huber et al., 2009; Migliati et al., 2009). However, the preparation procedure of the *Xenopus* oocytes before permeability assays renders this approach more adequate to validate previous identified inhibitors rather than to screen large libraries of compounds. Disclosure of novel inhibitors was also successfully performed in cultured cell lines selectively expressing AQP isoforms (Gao et al., 2006; Jelen et al., 2011; Wacker et al., 2013; Madeira et al., 2014b).

Control of AQP function by pH or specific inhibitors, can be screened through simple measurements of permeability and activation energy for water or solute transport after pre-incubation with the test compound and using the appropriate cell model assay. Human erythrocytes are known to express a large amount of AQP1 and AQP3 accountable for membrane permeability to water and glycerol, respectively (Campos et al., 2011), thus providing a simple screening assay to accurately evaluate inhibition or gating. Using the stopped-flow technique, human erythrocytes have been widely used to disclose AQP inhibitors (Niemietz and Tyerman, 2002; Yang et al., 2006; Martins et al., 2012, 2013). In addition, residues involved in phosphorylation (Maurel et al., 1995; Johansson et al., 1998; Kitchen et al., 2015) and also in any other gating mechanism can be identified *in silico* and validated *in vitro* by site-directed mutagenesis.

Levin et al. (2007) developed a simple screening method to identify inhibitors of AQP1 that involves measuring erythrocyte cell lysis using infrared light scattering. Erythrocyte expressing AQP1 and urea transporter (UT-B) were loaded with the urea analog acetamide, in the presence and absence of AQP1 inhibitors. In the absence of inhibitors, dilution of cells in a hypo-osmotic acetamide-free solution resulted in cell swelling and lysis. When AQP1 was inhibited, water influx was slower and dissipation of the osmotic gradient by acetamide efflux prevented cell lysis (Verkman, 2009).

A yeast phenotypic freeze–thaw assay was recently developed to screen for AQP inhibitors. Here, the survival of yeast cells exposed to freezing and thawing is dependent on their membrane water permeability, which is increased by heterologous AQP expression and impaired by inhibitors (To et al., 2015). Although promising as a high-throughput screening system, the identified inhibitors require further validation in a more robust biophysical assay.

Strategies to reveal AQP short-term regulation by membrane tension have been conceived. Membrane tension is related to the force needed to deform a membrane and is directly related to the product of pressure and radius. This means that to reach the same membrane tension, larger cells need much lower levels of pressure than smaller cells, i.e., the larger the cell the lower the pressure needed to trigger membrane deformation. Above a given pressure threshold cell membrane disrupts. Since these minimal pressures are difficult to manipulate experimentally, the effect of membrane tension on AQP activity in large mammalian cells is difficult to detect. Thus, assessment of AQP regulation by tension was achieved using membrane vesicles from mammalian tissues (Soveral et al., 1997a,c), walled yeast and plant cells (Soveral et al., 2008; Leitao et al., 2014), systems that are able to sustain high pressure gradients that induce different levels of tension without membrane rupture. A different approach was implemented by Ozu et al. (2005), using an emptied-out *X. laevis* oocyte as a diaphragm between two independent chambers. By allowing different levels of hydrostatic pressure, this procedure enabled to study human AQP1 gating by membrane tension (Ozu et al., 2013). Additionally, this system enables controlling the media composition on both compartments and detecting intracellular binding of inhibitors (Ozu et al., 2011).

*In silico* methods, in particular molecular docking and molecular dynamic simulations have been used to screen large libraries of compounds as candidate AQP inhibitors (Huber et al., 2009; Seeliger et al., 2013; Wacker et al., 2013). These methods are now considered essential for AQP drug discovery providing an educated guess for *in vitro* testing.

## Final remarks

In recent years, our understanding of the pathophysiology of water/glycerol balance disorders has increased enormously. AQPs have been progressively identified as key players in several physiological mechanisms and their dysfunction or aberrant expression implicated in disease, suggesting a great translational potential in aquaporin-based diagnostics and therapeutics.

Detecting AQP function, and characterizing their selectivity and mechanisms of gating is essential to establish AQP contribution to homeostasis, which is critical to health, and further identify dysfunctions that may lead to phenotypes such as those found in disease. In particular, the involvement of aquaglyceroporin-mediated glycerol transport in cell proliferation and adipocyte metabolism and its correlation with metabolic disorders and cancer, unveil the members of the aquaglyceroporin subfamily as promising therapeutic targets.

In spite of the numerous studies already available, several questions still remain open. Future research to untangle the biological relevance of AQPs as cause or consequence of the pathological condition ought to be conducted. Certainly more work is required to validate AQPs as drug targets, including careful analyses of the phenotypes of knockout models as well as pathophysiological studies in humans. In addition, the search for selective and non-toxic inhibitors that could be used as either chemical probes to detect AQP function in biological systems or as innovative therapeutic agents in a variety of disease states, should be encouraged.

These aspects can only be supported if the appropriate methods for functional analysis are conducted, including optimal technical/cell systems and accurate permeability evaluations. The use of *in silico* models together with well-designed experimental strategies are crucial for assessing AQP function and regulation, noticeably in the pursuit of innovative aquaporin-based drugs.

## Author contributions

AM discussed and wrote the manuscript. TM contributed to discussions and writing; GS proposed the outline, discussed, and supervised the writing.

### Conflict of interest statement

The authors declare that the research was conducted in the absence of any commercial or financial relationships that could be construed as a potential conflict of interest.
